# Cortical GABAergic Dysfunction in Stress and Depression: New Insights for Therapeutic Interventions

**DOI:** 10.3389/fncel.2019.00087

**Published:** 2019-03-12

**Authors:** Manoela V. Fogaça, Ronald S. Duman

**Affiliations:** Department of Psychiatry, Yale University School of Medicine, New Haven, CT, United States

**Keywords:** depression, stress, prefrontal cortex, GABA, somatostatin, parvalbumin, ketamine

## Abstract

Major depressive disorder (MDD) is a debilitating illness characterized by neuroanatomical and functional alterations in limbic structures, notably the prefrontal cortex (PFC), that can be precipitated by exposure to chronic stress. For decades, the monoaminergic deficit hypothesis of depression provided the conceptual framework to understand the pathophysiology of MDD. However, accumulating evidence suggests that MDD and chronic stress are associated with an imbalance of excitation–inhibition (E:I) within the PFC, generated by a deficit of inhibitory synaptic transmission onto principal glutamatergic neurons. MDD patients and chronically stressed animals show a reduction in GABA and GAD67 levels in the brain, decreased expression of GABAergic interneuron markers, and alterations in GABA_A_ and GABA_B_ receptor levels. Moreover, genetically modified animals with deletion of specific GABA receptors subunits or interneuron function show depressive-like behaviors. Here, we provide further evidence supporting the role of cortical GABAergic interneurons, mainly somatostatin- and parvalbumin-expressing cells, required for the optimal E:I balance in the PFC and discuss how the malfunction of these cells can result in depression-related behaviors. Finally, considering the relatively low efficacy of current available medications, we review new fast-acting pharmacological approaches that target the GABAergic system to treat MDD. We conclude that deficits in cortical inhibitory neurotransmission and interneuron function resulting from chronic stress exposure can compromise the integrity of neurocircuits and result in the development of MDD and other stress-related disorders. Drugs that can establish a new E:I balance in the PFC by targeting the glutamatergic and GABAergic systems show promising as fast-acting antidepressants and represent breakthrough strategies for the treatment of depression.

## Introduction

Major depressive disorder (MDD) is a recurring neuropsychiatric illness that is among the leading contributors to social and economic burden, affecting approximately one in five people in the United States (Kessler et al., 2005). The World Health Organization estimates that MDD will be the second leading cause of disability by 2020 (Reddy, 2010). Moreover, MDD induces a high level of personal suffering and suicidality, and also increases the risk of other comorbid medical conditions that can lead to further disability or death (Karch et al., 2009).

The current pharmacological treatment approaches set by national and international guidelines recommend the use of monoaminergic-based drugs, notably, serotonin reuptake inhibitors (SSRI), as first-line medications (Bauer et al., 2007). Although these drugs provide a significant therapeutic benefit, they still require weeks to months to induce an antidepressant response, and up to 33% of the patients are considered treatment resistant (i.e., fail to respond to two or more antidepressants). Also, the majority of patients experience recurrence after interrupting the treatment, and the adherence of patients to these medicines is relatively low, as they cause undesired side effects, including weight gain, sexual dysfunction, disruption of normal sleep patterns, memory deficits, and others. Moreover, most patients experience a worsening of symptoms in the first weeks of administration, which also contributes to the low treatment adherence (Trivedi et al., 2006).

Since the late 1980s, when the SSRIs were developed and the monoamine deficiency hypothesis of depression gained more support, there have been no considerable advances in pharmacological treatment for MDD. Considering the relatively low efficacy of monoaminergic drugs, there is an urgent need for development of novel medicaments that address current therapeutic limitations. Indeed, in recent years, a new class of fast-acting, efficacious antidepressants has emerged, showing immense promise in clinical and pre-clinical studies. Among them, ketamine, an NMDA receptor blocker, is the most studied due to its fast (within 2 h of administration) and sustained (up to 7 days) antidepressant effects (Berman et al., 2000; Zarate et al., 2006; Harmer et al., 2017). These glutamatergic-based drugs have shed light on yet unexplored avenues to explain the pathophysiology of depression, shifting efforts to the discovery and development of new classes of drugs. The accumulating evidence in the literature relating stress, GABA/glutamate deficits in the brain, and MDD, as well as the antidepressant efficacy of drugs that directly interact with these systems, have led to alternative hypotheses to explain the complex neurobiology of affective disorders that overcome inconsistences in monoaminergic theories.

In this review, we focus on the GABAergic deficit/imbalance hypothesis of MDD (Luscher et al., 2011; Fee et al., 2017), and elaborate this theory in the context of the glutamatergic hypothesis and the monoaminergic and neurotrophic deficits theories. We discuss the dysregulation of GABA neurotransmission and changes in specific GABAergic interneuron subtypes observed in MDD subjects and stress- or genetic-based animal models of depression. Although depression must be seen as a system-wide disorder, a broad range of GABA interneurons that orchestrate excitation–inhibition (E:I) balance in corticolimbic structures are located in the prefrontal cortex (PFC) and several studies point to this region as one of the primary brain regions involved in the pathophysiology of MDD. Indeed, multiple reports show a direct correlation between chronic stress and depression with decreased volume, synaptic atrophy/loss, and altered connectivity in the PFC (Duman et al., 2016). For this reason, we discuss in more detail evidence for cortical impairments in depression. Finally, based on recent findings, we will discuss how monoaminergic drugs can also modulate the GABAergic system and will explore novel non-monoaminergic fast-acting pharmacological approaches to treat MDD, including GABA_A_ and GABA_B_ receptor modulators (allosteric modulators, neurosteroids, agonists, and antagonists), NMDA receptors blockers (such as ketamine), and GABAergic interneurons-targeting neuropeptides.

## GABAergic System in the PFC

GABA is the major inhibitory mediator of cortical interneurons in the brain that serves to modulate a wide range of local neurotransmitter systems, most notably, the glutamatergic excitatory counterpart. By targeting specific somatic domains of neighboring glutamatergic principal neurons, GABA interneurons control the E:I balance in the PFC as well as the excitatory output to projecting areas, such as the amygdala, bed nucleus of stria terminalis, and dorsal raphe nucleus. Due to this network orchestration of firing patterns, cortical GABAergic interneurons play an essential role in mediating complex emotional and cognitive processes in the brain. GABA is synthesized from glutamate by glutamate decarboxylase enzymes (GAD65 and GAD67) and stored in vesicles through the vesicular GABA transporter (VGAT1 and 2). The GABAergic signal is terminated by rapid uptake of GABA to glial cells and presynaptic neurons through plasma membrane GABA transporters (GAT1-4).

One-third of all synapses in the central nervous system (CNS) connects via GABA interneurons, which comprise 20–30% of neocortical neurons and can be classified accordingly to their diverse morphological, electrophysiological, and molecular characteristics (Markram et al., 2004; Rudy et al., 2011; DeFelipe et al., 2013; Tremblay et al., 2016). The most common nomenclature segregates interneurons accordingly to their expression profile of neurochemical markers. Three major non-overlapping interneuron groups in the neocortex include those that express the calcium-binding protein parvalbumin (PV), the neuropeptide somatostatin (SST), and the ionotropic serotonin receptor 3 (Rudy et al., 2011). These neurons can further co-express other markers, such as the neuropeptides cholecystokinin (CCK), vasoactive intestinal peptide (VIP), and neuropeptide Y, as well as other calcium-binding proteins, such as calbindin and calretinin (Rudy et al., 2011). Characterization of distinct subtypes of interneurons helps to identify vulnerable subpopulations that could be relevant to different neuropsychiatric disorders. Specifically, PV and SST interneurons have been extensively studied in stress-related disorders. The most abundant subtype, PV, correspond to 40% of cortical GABA interneurons, and have chandelier or, most commonly, basket cell morphology. PV basket interneurons mainly control firing synchronization and spike timing of neighboring excitatory neurons by providing somatic fast-spiking inhibition to pyramidal cells (Markram et al., 2004; Ferguson and Gao, 2018). On the other hand, 25–30% of cortical interneurons express SST, which consist mainly of Marinotti cells with low-threshold regular spiking properties and an independent high basal firing activity. SST cells make synapses on the dendritic tufts of pyramidal cells but can also inhibit local PV interneurons (Markram et al., 2004; Urban-Ciecko and Barth, 2016). These distinct properties and sub-localization confer to both SST and PV interneurons different roles in the cortical microcircuit: while SST cells control the spiking inputs to pyramidal neurons, PV interneurons regulate the spiking outputs from pyramidal neurons to projecting brain areas.

GABA interneurons express two subtypes of GABA receptors: GABA_A_ and GABA_B_. The most prominent receptor, GABA_A_, is a ligand-gated Cl^-^ ion channel (ionotropic) and has been extensively characterized as the target of many psychotropic agents, including benzodiazepines, ethanol, and barbiturates. These receptors are mostly located post-synaptically and control fast synaptic inhibition. GABA_A_ receptors are tetrameric or pentameric in structure that are made up of multiple subunits (6α, 4β, and 3γ) in distinctive combinations that assemble together around a central chloride pore (Engin et al., 2018). GABA_B_ receptors are Gi-coupled receptors (metabotropic) and composed of a heterodimer of two homologous subunits: GABA_B1_ and GABA_B2_; they are mainly located at pre-synaptic sites, functioning as autoreceptors and inhibiting GABA release, although they can also be found post-synaptically (Cryan and Kaupmann, 2005). Given the broad spectrum of neuronal activity controlled by GABA interneurons, it is increasingly clear that imbalance in the GABAergic system and hence in the E:I balance can contribute to the pathophysiology of several psychiatric disorders, including MDD.

## Cortical Dysregulation of GABA Neurotransmission in Chronic Stress and Depression

Although the adaptive, innate stress response is essential for body homeostasis and survival, it is widely recognized that responses to sustained, chronic stress can become dysregulated and result in illness and abnormal behaviors. In the brain, chronic stress can produce changes in neurotransmitter function and appropriate neuroplasticity responses that could precipitate depression in humans and, therefore, has been extensively used as a rodent model for depression (Duman et al., 2016).

GABAergic neurons play an important role in the termination of stress response through regulation of the hypothalamus–pituitary–adrenal (HPA) axis, and disruption of this regulatory response contributes to the abnormal effects of chronic stress exposure. For example, chronic stress causes down-regulation of the transmembrane K-Cl cotransporter (KCC2), rendering GABA inputs ineffective to synaptic inhibition of the HPA axis (Hewitt et al., 2009). Moreover, deletion or mutation of the γ2 subunit of GABA_A_ receptors (heterozygous γ2 knockout: γ2+/-) result in reduced GABA_A_ receptor binding and consequent HPA axis hyperactivity, leading to anxiogenic and pro-depressive behaviors (Crestani et al., 1999; Chandra et al., 2005; Shen et al., 2010; Smith and Rudolph, 2012). A similar pro-depressive profile is found in α2 knockout mice (Vollenweider et al., 2011). Therefore, genetic modifications in GABA_A_ receptors subunits have been increasingly used as animal models to study the influence of GABAergic system in the pathophysiology of anxiety and depression, as well as pharmacological approaches that have therapeutic potential.

Considering that GABA receptors are highly expressed and GABAergic interneurons are abundant in the PFC and exert an important GABAergic inhibitory control over HPA axis activity (Diorio et al., 1993; Akana et al., 2001; Radley et al., 2009) it is conceivable that the PFC GABAergic system plays an essential role in emotional processing that is vulnerable to stress. In this respect, acute psychological stress (threat-of-shock condition) decreased approximately 18% of PFC GABA levels relative to a “safe” condition in healthy subjects (Hasler et al., 2010). In rodents, repeated immobilization stress increased GAD activity and GABA turnover, and reduced GABA levels in the frontal cortex (Otero Losada, 1988), an effect that was also reported after a 3-week of chronic mild stress (CMS) exposure (Shalaby and Kamal, 2009). In a learned helplessness paradigm, a model of depression, rats that failed to learn the shuttlebox task showed a 25% reduction of GABA_A_ receptors in cortical synaptoneurosomes (Drugan et al., 1989). Similar results were observed after other types of chronic stress, such as cold and isolation (Braestrup et al., 1979). A recent study reported that 9-weeks CMS exposure resulted in decreased cortical GABA_A_ receptor function, decreased release probability at peri-somatic GABAergic synapses, and reduced postsynaptic GABA_B_ receptor mediated inhibition in anhedonic rats, leading to higher excitability of pyramidal neurons (Czéh et al., 2018). Also, chronic unpredictable stress (CUS) or CMS exposure decreased innervation and function of GABAergic axons, and levels of GAD67, VGAT, and GAT3 in the PFC (Gilabert-Juan et al., 2013; Ma et al., 2016; Banasr et al., 2017). Besides chronic stress exposure of adult animals, there is also evidence that early life stress exposure impacts the GABAergic system later in life in the adult brain. Maternal separation stress and alteration of maternal care in the early (first weeks) postnatal period decreased expression of GABA_A_ receptors in the frontal cortex and other limbic areas, as well as induced anxiety and depressive-like behaviors in adulthood (Caldji et al., 2000, 2003).

Collectively, these data provide support for the hypothesis that stress causes major changes in the GABAergic system in the PFC that could result in abnormal behavioral and synaptic responses, including dendritic reorganization of interneurons (Gilabert-Juan et al., 2013), as well as alterations of electrophysiological respones (Northoff and Sibille, 2014; McKlveen et al., 2016), that results in defective output from pyramidal neurons to other brain areas. However, even though numerous reports suggest that chronic stress decreases GABA levels and function, other studies have reported opposite effects. Chronic immobilization stress induced a small increase in GABA_A_ receptor binding in the frontal cortex (Braestrup et al., 1979) and chronic social defeat stress increased GABA_A_-containing α5 subunit in the PFC and hippocampus of susceptible mice (Xiong et al., 2018). Likewise, chronic restraint stress (21 days) induced an increase in GABA_A_-α1 subunit mRNA expression in the mPFC but not α2, α3, α4, or γ2 (Gilabert-Juan et al., 2013). Moreover, McKlveen et al. (2016) found that CUS (14 days) increased the frequency of miniature inhibitory postsynaptic currents in the infralimbic area, as well as inhibitory appositions and terminals onto glutamatergic cells, suggesting a stress-induced enhancement of prefrontal inhibition. While difficult to reconcile, it is important to highlight that the results of stress studies may differ depending on the type and duration of the stressor, the GABA receptor subunit analyzed, and the specific subregions of the PFC studied.

In addition to these preclinical studies, there is accumulating evidence that dysfunction of the GABAergic system is associated with the pathophysiology of MDD and that normalization of GABA is associated with the remission of depressive symptoms (Godfrey et al., 2018). Pioneering studies showed that patients with depression have lower GABA levels in the plasma (Petty and Sherman, 1984) and the cerebrospinal fluid (CSF) (Gold et al., 1980; Gerner and Hare, 1981). Further studies extended this work through positron emission tomography (PET) imaging methods, which permits a direct and noninvasive quantification of GABA levels in the brain. These studies showed that GABA levels are reduced in unmedicated patients with MDD in several cortical areas, including the prefrontal (Hasler et al., 2007), occipital (Sanacora et al., 1999, 2004; Song et al., 2012), and anterior cingulate (ACC) cortices (Gabbay et al., 2012; Godfrey et al., 2018). Significant reduction in the ratio GABA/creatine + phosphocreatine was found in the ACC of female veterans with suicidal behavior (Prescot et al., 2018). Likewise, reduced GAD67 protein or gene expression were found in the dorsolateral PFC (dlPFC) and subgenual ACC (SgACC) of depressed patients (Karolewicz et al., 2010; Tripp et al., 2012), although other studies reported no significant effects (Sibille et al., 2011; Gilabert-Juan et al., 2012). Besides GABA levels, several studies reported decreased expression of GABA_A_ receptors subunit genes in MDD cortices, including decreased α1, α3, α4, γ1, β2, and ρ1 (Merali et al., 2004; Sequeira et al., 2007; Klempan et al., 2009; Luscher et al., 2011). However, there were also reports of increased expression of certain subunits, including α5, γ2, β3, and δ in MDD subjects (Merali et al., 2004; Choudary et al., 2005; Sequeira et al., 2007; Klempan et al., 2009) suggesting that different GABA_A_ receptor subunits may play distinct roles in the etiology of MDD.

Studies regarding the participation of GABA_B_ receptors in the pathophysiology of MDD have received less attention and, therefore, the literature remains unclear. Although GABA_B1_ and GABA_B2_ subunits were reported to be decreased in the lateral cerebellum of MDD subjects (Fatemi et al., 2011), no evidence was found for altered GABA_B_ receptor binding in the frontal cortex or hippocampus (Cross et al., 1988; Arranz et al., 1992). However, some variables should be considered as potential confounds in this study, as some of the MDD patients were taking antidepressants at the time of death, and in some cases there was a long post-mortem interval before tissue collection. Despite these limitations, it is notable that preclinical studies report that helpless rats showed decreased expression of GABA_B_ receptors in the frontal cortex (Martin et al., 1989), and GABA_B1_ subunit knockout animals displayed antidepressant-like responses in the forced swim test (Mombereau et al., 2004). Taken together, the results demonstrate that modulation of GABA_B_ receptors induces antidepressant effects (see the section “Conclusion and Future Directions”), and warrant additional studies with more cutting edge tools to further investigate the role of GABA_B_ receptors in depression and treatment response.

## GABA Interneuron-Related Deficits in Depression

Numerous studies suggest that the reduction in cortical GABA levels observed in MDD subjects and stressed rodents could not only result from decreased levels of the GABA synthetic enzymes GAD65/67, but could also result from a reduction in the density of specific GABA interneuron subpopulations (Table 1). MDD patients show a reduced volume of brain areas such as the PFC and hippocampus (MacQueen et al., 2008; Savitz and Drevets, 2009). Also, abnormalities in the GABAergic system in cortical areas can also robustly affect other brain regions. For example, low GABA levels in the ACC of MDD patients are associated with reduction in hippocampal volume (Abdallah et al., 2015). Reduced SST gene expression, mRNA, or protein levels were found in the CSF, SgACC, dlPFC, and amygdala of MDD subjects, and in the medial PFC (mPFC) and hippocampus of animals exposed to CUS (Rubinow et al., 1985; Rajkowska et al., 2007; Sibille et al., 2011; Tripp et al., 2011; Guilloux et al., 2012; Banasr et al., 2017). Interestingly, female MDD subjects show a more robust reduction in SST expression than males (Sibille et al., 2011; Tripp et al., 2011, 2012; Guilloux et al., 2012), suggesting that SST could be related to the twofold greater incidence of MDD in females (Kuehner, 2017). Although SST expression in MDD subjects and chronically stressed animals is decreased, Gilabert-Juan et al. (2013) reported dendritic hypertrophy of Martinotti cells (which includes SST-expressing interneurons) in the mPFC of mice exposed to chronic restraint stress, without changes in spine density.

**Table 1:** Studies of GABAergic interneuron subtypes in MDD and animal models of depression.

Cell	Species	Model	Brain region	Gender	Effect	Reference
SST	Human	MDD	SgACC	M/F	↓ SST protein expression, SST mRNA, and SST-related genes	Tripp et al., 2011, 2012
			Dorsolateral PFC	M/F	↓ SST protein and mRNA expression	Sibille et al., 2011
			Amygdala	F	↓ SST protein expression, SST mRNA, and SST-related genes	Guilloux et al., 2012, Douillard-Guilloux et al., 2017
	Mouse	SST KO	Cingulate cortex	M/F	↑ Emotionality and plasma corticosterone levels ↓ BDNF, GAD67, and cortistatin gene expression ↓ EIF2 signaling in SST interneurons	Lin and Sibille, 2015
		BDNF^+/-^ and BDNF^KIV^ mice	Cingulate cortex	M/F	↓ SST gene expression	Tripp et al., 2012
		DREADD-hM4Di DTA-AAV ± CUS (6 weeks)	mPFC	M/F	– Acute CNO (30 min): anxiogenic and pro-depressive responses – Chronic CNO (3 weeks): anxiolytic and antidepressant responses – Anxiolytic and antidepressant responses in baseline and CUS animals	Soumier and Sibille, 2014
		GABA-γ2 subunit deletion in SST interneurons (SSTCre:γ2^f/f^)	Forebrain	M/F	– Anxiolytic and antidepressant responses ↓ eEF2 phosphorylation	Fuchs et al., 2017
		Chronic restraint stress (21 days)	mPFC	M	– Dendritic hypertrophy of Martinotti cells; no spine density alteration	Gilabert-Juan et al., 2013
	Rat	CUS (7 weeks)	Orbitofrontal cortex	M	– No alteration in SST neuron density	Varga et al., 2017
		CUS (36 days)	PFC	M	↓ SST mRNA	Banasr et al., 2017
		CMS (9 weeks)	mPFC	M	– No alteration in SST neuron number	Czéh et al., 2018
		CMS (9 weeks)	Hippocampus	M	↓ SST neuron number in anhedonic and resilient rats	Czéh et al., 2015
PV	Human	MDD	SgACC	M/F	↓ PV gene expression	Tripp et al., 2012
		MDD	ACC	M/F	– No alteration in PV neuron density	Beasley et al., 2002
		MDD (majority suicide victims)	dlPFC	M/F	– No alteration in PV mRNA	Sibille et al., 2011
					– No alteration in PV neuron density (area 9)	Rajkowska et al., 2007
			Orbitofrontal cortex	M/F	– Modest PV neuron density reduction in area 47 (*p* = 0.05)	Rajkowska et al., 2007
	Mouse	BDNF^+/-^ and BDNF^KIV^ mice	Cingulate cortex	M/F	– No alteration in PV gene expression	Tripp et al., 2012
		CMS (2 or 4 weeks)	mPFC	F	↑ PV mRNA and PV neuron number	Shepard et al., 2016; Shepard and Coutellier, 2018
		Learned helplessness DIO-hM4Di: CNO administered 30 min prior LH sections/test		M	↓ Excitatory synaptic transmission onto PV interneurons in mice showing helplessness ↑ Helplessness	Perova et al., 2015
		DREADD-hM3D-Gq: CNO 30 min prior test	Dentate gyrus	M	– No effect in depression-like behavior (tail suspension test); anxiolytic and increased fear extinction	Zou et al., 2016
		GAD1 knockdown in PV interneurons (PV/GAD1 Ig)	Multiple brain regions	M	– Sensoriomotor gating deficits, increased novelty seeking, and decreased fear extinction	Brown et al., 2015
	Rat	Chronic social isolation (21 days)	mPFC	M	↓ PV neuron number	Todorovic et al., 2019
		CUS (16 days) CMS (8 weeks)			– No alteration in PV neuron density and neuropil	Zadrozna et al., 2011
		CMS (9 weeks)			↓ PV neuron number in anhedonic rats	Czéh et al., 2018
		CUS (36 days)	PFC	M	– No alteration in PV protein expression	Banasr et al., 2017
		CUS (7 weeks)	Orbitofrontal cortex	M	– No alteration in PV neuron density	Varga et al., 2017
		CMS (9 weeks) Chronic social isolation (21 days)	Hippocampus	M	↓ PV neuron number	Filipovic et al., 2013; Czéh et al., 2015; Csabai et al., 2017
		CUS (16 days) CMS (8 weeks)			– No alteration in PV neuron density	Nowak et al., 2010
CB	Human	MDD (majority suicide victims)	Dorsolateral PFC (Brodmann’s 9)	M/F	↓ CB neuron density and somata size	Rajkowska et al., 2007
			Occipital cortex (Brodmann’s 17)		↓ CB neuron density – No alteration in cell size	Maciag et al., 2010
	Rat	CUS (36 days)	PFC	M	– No alteration in CB protein expression	Banasr et al., 2017
		CUS (9 weeks)	mPFC	M	– No alteration in CB neuron number	Czéh et al., 2015
		CUS (16 days) CMS (8 weeks)			↓ CB neuron and neuropil densities ↑ CB neuron and neuropil densities	Zadrozna et al., 2011
		CUS (7 weeks)	Orbitofrontal cortex	M	↓ CB neuron density	Varga et al., 2017
		CMS (9 weeks)	Hippocampus	M	– No alteration in CB neuron number	Czéh et al., 2018
		CMS (8 weeks) CUS (16 days)			↓ CB neuron density	Nowak et al., 2010
NPY	Human	MDD	SgACC	M/F	↓ NPY gene expression	Tripp et al., 2012
		Suicide	Frontal cortex and caudate nucleus	M/F	↓ NPY levels	Widdowson et al., 1992
		MDD and suicide	Frontal cortex	M/F	– No alteration in NPY levels	Ordway et al., 1995
	Mouse	BDNF^+/-^ and BDNF^KIV^ mice	Cingulate cortex	M/F	↓ NPY gene expression	Tripp et al., 2012
	Rat	CUS (36 days)	PFC	M	↓ NPY mRNA	Banasr et al., 2017
		CMS (9 weeks)	mPFC	M	↑ NPY neuron number in CMS resilient rats	Czéh et al., 2018
		CUS (7 weeks)	Orbitofrontal cortex	M	↑ NPY neuron density in CUS resilient rats	Varga et al., 2017
		CMS (9 weeks)	Hippocampus	M	↓ NPY neuron number	Czéh et al., 2015
		Flinders sensitive line			↓ NPY levels	Caberlotto et al., 1999
				F	↓ NPY mRNA	Melas et al., 2012
CR	Human	MDD	SgACC	M	↓ CALB2 gene expression	Tripp et al., 2012
			Dorsolateral PFC	M/F	– No alteration in CR mRNA	Sibille et al., 2011
	Mouse	BDNF^+/-^ and BDNF^KIV^ mice	Cingulate cortex	M/F	– No alteration in CR gene expression	Tripp et al., 2012
	Rat	CUS (36 days)	PFC	M	– No alteration in CR protein expression	Banasr et al., 2017
		CMS (9 weeks)	mPFC	M	↓ CR neuron number in anhedonic and resilient rats	Czéh et al., 2018
		CUS (7 weeks)	Orbitofrontal cortex	M	– No alteration in CR neuron density	Varga et al., 2017
		CMS (9 weeks)	Hippocampus	M	↓ CR neuron number	Czéh et al., 2015
CCK	Rat	CUS (36 days)	PFC	M	– No alteration in CCK protein expression	Banasr et al., 2017
		CMS (9 weeks)	mPFC	M	↓ CCK neuron number	Czéh et al., 2018
		CUS (7 weeks)	Orbitofrontal cortex	M	↑ CCK neuron density in resilient rats	Varga et al., 2017
		CMS (9 weeks)	Hippocampus	M	– No alteration in CCK neuron number	Czéh et al., 2015; Csabai et al., 2017


*SST, somatostatin; PV, parvalbumin; CB, calbindin; CR, calretinin; NPY, neuropeptide Y; CCK, colecistokinin; MDD, major depressive disorder; CUS, chronic unpredictable stress; CMS, chronic mild stress; PFC, prefrontal cortex; mPFC, medial prefrontal cortex; M, male; F, female; BDNF^*KIV*^, targeted disruption of BDNF exon IV.*

The association between SST interneurons and the pathophysiology of MDD has been more directly supported through pharmacological and genetic manipulations in rodents. Mice lacking SST (SST-KO) exhibited increased anxiety- and depressive-like behaviors; elevated basal plasma corticosterone; and reduced BDNF, GAD67, and cortistatin genes expression (Lin and Sibille, 2015). Disinhibition of SST interneurons by deletion of GABA_A_-containing γ2 subunit in SST neurons (SSTCre:γ2^f/f^) resulted in enhanced inhibitory input to pyramidal cells in the hippocampus and cingulate cortex, and consequently produced anxiolytic- and antidepressant-like phenotypes (Fuchs et al., 2017). Interestingly, acute chemogenetic-induced inhibition of SST interneurons in the mPFC promoted anxiety and depressive-like responses, whereas chronic silencing or chemical ablation had the opposite effect (Soumier and Sibille, 2014). Furthermore, mice with constitutive, heterozygous deletion of the BDNF gene (BDNF+/-) or with targeted disruption of exon IV (BDNF^KIV^), causing a reduction or blockade of activity-dependent BDNF expression and depressive-like behaviors, showed reduced SST and NPY gene expression in the cingulate cortex (Tripp et al., 2012).

Somatostatin co-localizes with calbindin and NPY, and these neuropeptides have also been implicated in mood disorders. Reductions in calbindin and NPY markers were found in the frontal cortex of MDD patients (Widdowson et al., 1992; Rajkowska et al., 2007; Maciag et al., 2010; Tripp et al., 2011, 2012), as well as in the PFC and hippocampus of rats submitted to different rodent models of depression, including CUS, BDNF mutant mice, or the Flinders sensitive line of rat (Caberlotto et al., 1999; Nowak et al., 2010; Zadrozna et al., 2011; Melas et al., 2012; Tripp et al., 2012; Czéh et al., 2015, 2018; Banasr et al., 2017; Varga et al., 2017). However, other studies failed to detect statistical differences in chronic stress models (Czéh et al., 2015, 2018; Banasr et al., 2017). Additionally, NPY neuronal density was increased in the orbitofrontal cortex and mPFC (IL) of rats that were considered resilient to CUS behavioral effects, but unchanged in anhedonic animals (Varga et al., 2017; Czéh et al., 2018). In depressed patients, the levels of NPY in the CSF or plasma were inversely correlated with anxiety symptoms and with attempted suicide (Widerlov et al., 1988; Westrin et al., 1999). Other markers, such as CCK and calretinin, seem not to be consistently affected in stress models or MDD (Sibille et al., 2011; Zadrozna et al., 2011; Tripp et al., 2012; Banasr et al., 2017; Csabai et al., 2017), notwithstanding a study reporting decreased immunoreactivity of these peptides in the mPFC of rats submitted to CMS (Czéh et al., 2018).

Although several studies have failed to detect robust differences in the expression of PV in rodent models and MDD subjects (Beasley et al., 2002; Cotter et al., 2002; Rajkowska et al., 2007; Nowak et al., 2010; Sibille et al., 2011; Zadrozna et al., 2011; Banasr et al., 2017), there are reports that PV interneurons contribute to regulation of E:I within the PFC that influences emotional responses (Perova et al., 2015; Ferguson and Gao, 2018). One study has reported a decrease in PV gene expression in SgACC post-mortem tissues of depressed patients (Tripp et al., 2012), and a modest reduction in PV immunoreactivity was found in the orbitofrontal cortex (*p* = 0.05, Brodmann area 47) (Rajkowska et al., 2007). In rodents, CMS caused a reduction in PV neuron number in the mPFC of anhedonic rats, whereas SST density was unchanged (Czéh et al., 2018). A decrease in PV cell number was also found in rats submitted to chronic social isolation (Todorovic et al., 2019). By contrast, there was a report of increased PV expression in the PFC of female mice after 2 weeks of CUS (Shepard et al., 2016; Shepard and Coutellier, 2018). Also, mice that showed helplessness behavior in response to inescapable stress exposure showed a reduction in excitatory synaptic transmission onto PV interneurons in the mPFC, and selective chemogenetic inactivation of PV cells further increased helplessness responses (Perova et al., 2015).

However, other genetic approaches have reported complex behavioral changes. Knockdown of the *Gad1* transcript specifically in PV interneurons (*Pvalb*/*Gad1* Tg) produced a decrease in PV-induced GABAergic activity in multiple brain regions, leading to sensoriomotor gating deficits, increased novelty seeking, and decreased fear expression (Brown et al., 2015). Besides the PFC, PV cells located in the hippocampus also play an important role in the modulation of affective behaviors as well as memory. Acute activation of PV interneurons in the dentate gyrus of the hippocampus through DREADD-hM3D-Gq virus and CNO administration (30 min) did not affect depressive-like behavior in the tail suspension test, but produced anxiolytic-like responses and increased fear extinction (Zou et al., 2016). Also, CMS or chronic social isolation induced a decrease in PV-immunoreactive cells in the hippocampus, whereas CCK and calbindin expression remained unchanged (Filipovic et al., 2013; Czéh et al., 2015; Csabai et al., 2017). Thus, divergence in the responses observed among these studies in the literature highly are related to the brain region studied, the experimental protocol (timing, stress paradigms), and sex differences. Also, it is noteworthy that PV interneurons are under inhibitory control of other interneuron populations, such as SST, making the resultant responses even more complex.

Taken together, the studies mentioned highlight the complexity of segregating the GABAergic system into different subclasses of interneurons to study the pathophysiology of depression, but demonstrate the importance of understanding how these cells locally interact and integrate diverse neurocircuits that control affective behavioral responses.

## The GABAergic System as a Therapeutic Target for the Treatment of MDD

Although extensive efforts have been conducted to develop new therapeutic interventions, the current pharmacological treatment approaches still recommend the use of SSRIs as first-line medications for the treatment of MDD. These drugs, along with other classic antidepressants, such as tricyclics and monoamine oxidase inhibitors, primarily facilitate monoaminergic systems, including 5-HT and norepinephrine. However, the emergence of fast-acting antidepressants, notably ketamine, provide evience for other neurotransmitter systems for the treatment, as well as pathophysiology of MDD. Evidence that normalization of GABA-mediated E:I imbalance in the PFC is a shared mechanism of action between different classes of antidepressants, providing further support for the involvement of GABAergic dysfunction in the etiology of MDD. In this section, we will review the literature showing how first-line monoaminergic antidepressants and rapid-acting agents can influence the GABAergic system. We will discuss how fast-acting antidepressants provide a new understanding of the pathophysiology of depression, leading to connections between the glutamatergic, GABAergic, and neurotrophic hypotheses of depression. Finally, we will discuss the antidepressant potential of agonists, antagonists, or allosteric modulators of GABA_A_ and GABA_B_ receptors, as well as neuropeptides that target specific subpopulations of GABA interneurons and cannabinoid agents.

### Classic Monoaminergic Antidepressants: Effects Beyond Monoamines

The correlation between GABA deficits in the brain, stress, and MDD became more evident with investigations showing that SSRIs, electroconvulsive therapy, and transcranial magnetic stimulation normalize the reduction in cortical and plasmatic GABA levels, as well as in GAD67 expression in MDD subjects and rodents subjected to chronic stress (Sanacora et al., 1999, 2004; Bhagwagar et al., 2004; Goren et al., 2007; Kucukibrahimoglu et al., 2009; Karolewicz et al., 2010; Dubin et al., 2016). Besides decreased GABA levels, MDD patients and chronically stressed animals have reduced levels of allopregnanolone (brain and plasma), an endogenous neurosteroid that acts as a GABA_A_ receptor positive allosteric modulator (discussed in more detail below). This deficit was reversed by chronic administration of SSRIs such as fluoxetine (Uzunov et al., 1996; Romeo et al., 1998; Uzunova et al., 1998, 2004; Strohle et al., 1999; Dong et al., 2001; Guidotti et al., 2001; Pinna et al., 2006, 2009); interestingly, *in vitro* evidence suggests that SSRIs can directly interact with the enzymes involved in neurosteroid synthesis (Griffin and Mellon, 1999). Chronic treatment with the classic monoaminergic antidepressant desipramine, but not fluoxetine, also normalized the elevated serum corticosterone levels and the pro-depressive behaviors of γ2+/- mice (Shen et al., 2010). In this same study, subchronic treatment with desipramine had no effect (Shen et al., 2010), suggesting that this drug acts over time to balance GABAergic inhibition deficits. In another study, chronic fluoxetine treatment induced pro-depressive and anxiogenic-like effects in γ2+/- mice (Benham et al., 2017), pointing to a requirement of GABA_A_-containing γ2 subunit in the antidepressant effect of SSRIs. Interestingly, studies reported that fluoxetine can act directly as an allosteric modulator of GABA_A_ receptors (Robinson et al., 2003).

Indeed, direct interactions between the GABAergic and serotoninergic systems in the raphe nucleus and cortical regions have been reported (Celada et al., 2001; Puig et al., 2004; Santana et al., 2004; Llado-Pelfort et al., 2012). In the PFC, both pyramidal glutamatergic neurons and GABAergic interneurons, notably PV positive cells, express serotoninergic receptors (mainly 5HT_1A_ and 5HT_2A_) (Santana et al., 2004; Celada et al., 2013). 5HT_1A_ receptor agonists, such as 8-OH-DPAT, have a preferential action on GABA interneurons, resulting in pyramidal neuron disinhibition and enhancement of cell firing in PFC and targeted subcortical structures, such as the ventral tegmental area (Llado-Pelfort et al., 2012). On the other hand, the excitability of pyramidal neurons in the mPFC can be inhibited by activation of GABA interneurons through 5HT_3_ receptors (Puig et al., 2004). In this regard, multimodal drugs that are high affinity 5HT_3_ receptor antagonists, such as vortioxetine, show antidepressant efficacy in clinical studies and have been used as atypical antidepressants to treat MDD (Thase et al., 2016; Artigas et al., 2018). Moreover, the majority of serotoninergic cell bodies in the raphe nucleus express GABA_B_ receptors, which control serotoninergic cell firing as well as the release of monoamines in other brain regions (Bowery et al., 1980; Abellan et al., 2000; Serrats et al., 2003). The antidepressant-like effects of the GABA_B_ antagonist CGP56433A were abolished by prior treatment with a tryptophan hydroxylase inhibitor, which depletes serotonin levels (Slattery et al., 2005). Additionally, several different monoaminergic antidepressants increase GABA_B_ receptor binding and function in the rat frontal cortex (Lloyd et al., 1985; Gray and Green, 1987).

### Fast-Acting Glutamatergic Antidepressants: Is It All Glutamate?

In recent years, the mechanisms underlying the actions of ketamine have been extensively studied because of its rapid (within hours), sustained (up to 7 days), and efficacious effects (effective in patients considered treatment resistant) (Berman et al., 2000; Zarate et al., 2006). Related agents, including ketamine stereioisomers and metabolites, have also demonsrated rapid effects in rodent models. These drugs share the ability to influence, directly or indirectly the enhancement of glutamatergic signaling in the brain, promoting post-synaptic AMPA-mediated calcium influx that leads to BDNF release by pyramidal neurons (Lepack et al., 2014, 2016; Zhou et al., 2014). Extracellular BDNF, in turn, activates TrkB receptors in the membrane, resulting in stimulation of intracellular signaling cascades, including Akt, eukaryotic elongation factor 2 kinase (eEF2K), and mTORC1 that results in synaptic actions that contribute to antidepressant behavioral responses (Li et al., 2010; Autry et al., 2011; Duman et al., 2016).

The molecular and cellular mechanisms underlying the rapid enhancement of glutamatergic signaling in the PFC by ketamine have been of particular interest. One hypothesis is that ketamine first targets NMDA receptors specifically located in cortical interneurons, notably, SST and PV subtypes. Because these GABA inhibitory neurons are tonic firing they would be more sensitive to antagonist blockade as tonic activity would remove the Mg^2+^ block of the NMDA receptor allowing ketamine to enter the channel pore and block further activation of Ca^2+^ entry (Fee et al., 2017; Ghosal et al., 2017). Blockade of GABAergic interneuron firing would thereby decrease GABA release, resulting in disinhibition of excitatory pyramidal neurons and subsequently produce a glutamate burst that could drive activity dependent synaptic plasticity (Duman et al., 2016). An alternative hypothesis is that ketamine acts directly on pyramidal neurons to block resting state NMDA receptor activity driven by spontaneous glutamate release that produces synaptic changes via deactivation of eEF2K, resulting in increased synthesis of synaptic proteins (Autry et al., 2011). These two theories may not be mutually exclusive, although it is difficult to explain how NMDA receptors would be at resting levels in the presence of a known glutamate burst (Moghaddam et al., 1997). In either case, there is an increase in synaptic protein synthesis that underlies long-lasting changes (approximately 1 week) that correspond to the time course for the antidepressant behavioral actions of ketamine. It is also possible that more long-lasting ketamine metabolites contribute to the sustained actions of ketamine (Zanos et al., 2016; Fukumoto et al., 2019). The glutamate burst produced by ketamine appears to be contradictory with evidence of elevated glutamate levels in the brains of MDD subjects (Stone et al., 2012), although other studies have reported no significant differences (Valentine et al., 2011; Taylor et al., 2012). However, it is important to note that the ketamine-induced burst of glutamate is transient, lasting approximately 1 h, and then levels return to control (Moghaddam et al., 1997). Although transient, the glutamate burst results in activity-dependent synaptic changes that are long-lasting.

Although much of the current work has focused on glutamate synaptic changes in the actions of ketamine, there is also increasing evidence that GABA alterations contribute to the ketamine response, by reestablishing E:I balance in the PFC via homeostatic self-tuning adaptations. This local reorganization could influence microcircuits in target regions by reestablishing firing patterns, and thereby promoting antidepressant effects. This idea is supported by recent evidence that the fast antidepressant effects of ketamine are accompanied by a robust increase in GABA levels in the mPFC of MDD patients (Milak et al., 2016) and in the ACC of rats subjected to CUS (Perrine et al., 2014), although another study failed to detect differences in the occipital cortex (Valentine et al., 2011). One possibility for these discrepancies, in addition to the different cortical subregions analyzed, is the timepoint at which MRS data were collected. Whereas one study was conducted during ketamine infusion (Milak et al., 2016), the other was carried out after ketamine (Valentine et al., 2011); by the end of the infusion, it was shown in the former study that the increase in amino acid responses was no longer detectable (Milak et al., 2016).

Also, a SPECT study reports that S-ketamine administration leads to alterations of GABA_A_ receptor binding in the dorsomedial PFC of healthy subjects (Heinzel et al., 2008). Likewise, studies in cultured murine neurons provide evidence that ketamine increases the activity of extrasynaptic GABA_A_ receptors in the cortex and hippocampus (Wang et al., 2017). Combined administration of sub-effective doses of muscimol, a potent and selective agonist of GABA_A_ receptors, and ketamine, produced antidepressant-like effects in female mice (Rosa et al., 2016). In this same study, the antidepressant effects of ketamine were blocked by the GABA_B_ agonist baclofen, suggesting that the antidepressant actions of ketamine could involve activation of GABA_A_ and blockade of GABA_B_ receptors (Rosa et al., 2016). In support of glutamatergic and GABAergic interactions in ketamine responses is data showing that a single dose of the ketamine induced antidepressant-like effects and normalized the glutamatergic deficits, including reduced cell surface NMDA and AMPA receptor levels and impaired synaptic function in the hippocampus and mPFC of γ2+/- mice (Ren et al., 2016). Moreover, ketamine potentiated pre- and post-synaptic GABAergic synapses selectively in the ACC of these animals (Ren et al., 2016). In addition, we have found that a single dose of ketamine increases markers of GABA in the PFC, including increased levels of VGAT, GAD, and gephyrin (Ghosal et al., 2018, SfN abstract). Thus, although more studies are needed to clarify how ketamine modulates the GABAergic system, the current evidence indicates that ketamine enhances GABA levels/function in the brain as well as GABA_A_ receptors activity.

**FIGURE 1:**
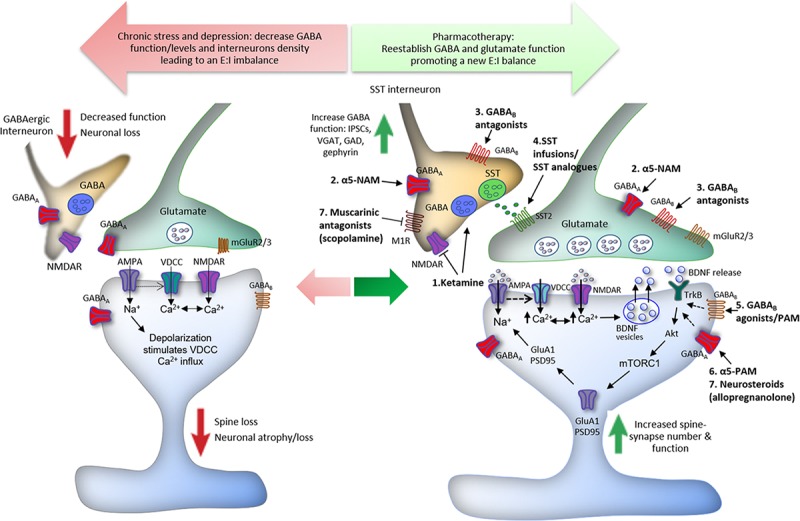
Proposed mechanisms underlying the action of ketamine and GABA-related drugs in the reestablishment of cortical excitatory–inhibitory (E:I) balance. Chronic stress induces spine loss and dendritic atrophy in pyramidal glutamatergic cells, and decreased GABAergic interneuron markers, leading to a reduction in the levels and function of GABA in the PFC. GABAergic dysfunction disturbs the optimal E:I balance in the brain and compromises the integrity of neurocircuits, contributing to the development of major depressive disorder (MDD) and other stress-related disorders. The E:I imbalance can be reversed by drugs via different GABA-related mechanisms. (1) Low doses of ketamine induce a glutamate burst in the PFC via blockade of NMDA receptors located in GABAergic interneurons; the tonic firing of these interneurons, notably parvalbumin (PV) and somatostatin (SST), is driven by NMDA receptors that are more sensitive to ketamine because of activity dependent of the Mg^2+^ block. This leads to disinhibition of pyramidal neurons causing activation of post-synaptic AMPA receptors; this in turn induces neuronal depolarization and activation of voltage-dependent Ca^2+^ channels (VDCCs). The enhancement of intracellular Ca^2+^ influx leads to BDNF release and stimulation of TrkB receptors, which activates mTORC1 signaling inducing protein synthesis required for the formation of new spines and synaptic plasticity. Ketamine also facilitates GABA-mediated effects, increasing IPSCs, VGAT, GAD, and gephyrin in the PFC, reversing the GABA deficits caused by chronic stress exposure. (2) Likewise, α5-GABA_A_ negative allosteric modulators (α5-NAMs) and (3) GABA_B_ receptors antagonists, probably located in GABAergic interneurons, enhance glutamatergic neurotransmission and produce ketamine-like effects. (4) Infusions of SST or SST analogs into limbic brain regions produce antidepressant-like effects through activation of SST2 receptors. (5) Finally, activation of post-synaptic GABA_B_ receptors by agonists or positive allosteric modulators (PAMs), as well as activation of α5-GABA_A_ by PAMs, and other GABA_A_ subunits by neurosteroids, notably allopregnanolone (6), can also recruit BDNF expression and signaling that could contribute to antidepressant responses (dashed arrows).

In addition, it was reported that fast-acting agents, such as Ro-25-6981, a GluN2B-selective NMDA receptor antagonist, induce antidepressant effects by promoting GABA_B_ receptor surface expression and increasing postsynaptic GABA_B_-mediated resting L-type calcium channel activity, resulting in an increased intracellular calcium, recruitment of BDNF/mTORC1 pathways, and protein synthesis (Workman et al., 2013, 2015). Accordingly, MDD and suicide patients have decreased levels of blood and brain BDNF levels and transcripts (Dwivedi et al., 2003; Shimizu et al., 2003; Kim et al., 2007; Guilloux et al., 2012; Banerjee et al., 2013); reduced BDNF levels in blood were absent in patients taking antidepressants (Shimizu et al., 2003). However, while monoaminergic antidepressants take weeks to modulate neurotrophic factor expression, the rapid elevation in BDNF “release” and signaling by ketamine is shared by other fast-acting agents, such as the non-selective muscarinic receptor antagonist scopolamine, the NMDA receptor modulator GLYX-13 (rapastinel), the ketamine metabolite (2R,6R)-Hydroxynorketamine [(2R,6R)-HNK], and the mGlu2/3 receptor antagonist LY341495 (Liu et al., 2012; Lepack et al., 2016; Ghosal et al., 2018; Kato et al., 2018) and may explain, at least in part, the fast versus slow response rates of these agents. Moreover, *in vitro* and *in vivo* studies suggest that BDNF induces antidepressant-like effects via increased phosphorylation of γ2 subunit, resulting in an increase of GABA_A_ receptor accumulation and stability in the cell surface, and in an enhancement of synaptic inhibition efficacy in the hippocampus and PFC (Jovanovic et al., 2004; Vithlani et al., 2013). Thus, the upregulation of GABA_B_ receptors induced by NMDA receptor blockade and consequent activation of BDNF/mTORC1 signaling, as well as the role of BDNF on GABA_A_ receptors phosphorylation and enhancement of GABAergic mIPSC amplitude and frequency, could be a link associating the GABA/glutamate balance deficits to the neurotrophic theory of depression (Figure 1).

### GABA Ligands

#### GABA_A_-α2 Positive Allosteric Modulators

Deletion studies of α2-containing GABA_A_ subunit demonstrate a role of these receptors in depressive behaviors and suggest that agonists or positive modulators could produce antidepressant effects. Benzodiazepines (BZD), GABA_A_ positive allosteric modulators developed in the 1950s, are one of the most widely used thereapeutic agents for the treatment of psychiatric disorders, due to anxiolytic actions at the GABA_A_-α2 receptor and hypnotic effects at the GABA_A_-α1 (Mohler et al., 2002). However, the efficacy of classical BZD monotherapy for the treatment of MDD has not been consistently reported, in part due to methodological confounds (i.e., small sample size, variable duration of treatment, and cotreatment with antidepressant agents), as well as high comorbidity with anxiety disorders (Pehrson and Sanchez, 2015). However, the triazolobenzodiazepine alprazolam was shown to induce significant antidepressant effects similar to tricyclic drugs in several meta-analysis studies (Jonas and Cohon, 1993; Birkenhager et al., 1995; Petty et al., 1995; van Marwijk et al., 2012). This antidepressant potential has been attributed to its differential chemical structure formed by a triazol ring fused to the diazepine ring. Recently, selective agonists or positive modulators of GABA_A_-containing α2/α3 subunits, such as TPA023 and eszopiclone, have been developed and are proposed as potential antidepressants (Atack, 2011; Atack et al., 2011; Vollenweider et al., 2011). When co-administered with SSRIs, eszopiclone, a preferential α2/α3-GABA_A_ positive modulator, induced a faster onset of efficacy and greater treatment response, suggesting a synergistic effect (Fava et al., 2006, 2011; Krystal et al., 2007). Although it is still unclear if increasing the activity of GABA_A_ receptor is effective in relieving depression symptoms, given that some BZD seem to have greater antidepressant efficacy (i.e., alprazolam *versus* diazepam), a more thorough understanding of the role of different subunits/subtypes of GABA_A_ receptors could result in the development of more selective and efficacious antidepressant drugs.

#### GABA_A_-α5 Negative Allosteric Modulators

Recently, a new class of fast-acting antidepressants that specifically target the GABA_A_-containing α5 subunit has emerged (Atack et al., 2009; Zanos et al., 2017; Xiong et al., 2018). These receptors were shown to be up-regulated in the cortex and hippocampus of depressed patients and stressed mice (Matsumoto et al., 2007; Xiong et al., 2018). Interestingly, preclinical studies have demonstrated that both positive and negative allosteric modulators of GABA_A_-α5 receptors produce rapid antidepressant-like effects or prevent the behavioral responses induced by chronic stress (Zanos et al., 2017; Xiong et al., 2018). This apparent discrepancy could be due to ketamine-like induction of a glutamate burst for negative modulators and GABA_A_ receptor dependent effects of positive modulators. In one study, MRK-016, a negative allosteric modulator of GABA_A_-α5 receptors and partial inverse agonist of the BZD-binding site, produced a transient increase in electroencephalogram γ power, similar to ketamine. These effects of MRK-016 were abolished by NBQX, an AMPA-type glutamate receptor antagonist, suggesting a fast recruitment of the glutamatergic system and activity-dependent effects. Importantly, perhaps because of the restricted distribution of GABA_A_-α5 receptors in the brain (mainly in the cortex and hippocampus), MRK-016 did not induce the typical ketamine-like side effects indicative of psychotomimetic or cognitive impairment (Zanos et al., 2017). Unfortunately, further development of this compound has been discontinued because of low tolerability in elderly subjects (Atack et al., 2009; Atack, 2011; Rudolph and Knoflach, 2011). Another GABA_A_-α5 negative allosteric modulator, L-655,708, also restored alterations in hedonic behaviors induced by chronic stress and the excitatory synaptic strength in the CA1 region of the hippocampus (Fischell et al., 2015). A single dose of L-655,708 increased the expression of the GluA1 subunit of the AMPA receptor, suggesting that, similar to MRK-016, it produces an indirect potentiation of excitatory synapses. Other GABA_A_-α5 negative modulators have been tested as cognitive enhancers in clinical trials, but these agents have not been tested for effectiveness in depression (Rudolph and Knoflach, 2011).

Surprisingly, similar antidepressant-like effects were found after acute and chronic enhancement of α5-GABA_A_ activity by a positive modulator; however, this response was restricted to female mice and absent in males, suggesting sex-effects (Piantadosi et al., 2016). Since the behavioral sex differences could not be explained by differential pharmacokinetic effects (i.e., different brain concentrations), it is possible that α5-GABA_A_ positive modulators interact with steroid hormones to produce an antidepressant response. Indeed, GABA_A_ receptor subunits, such as δ, are highly sensitive to neurosteroids (see next section) and are differentially modulated across the estrous cycle (Maggi and Perez, 1986; Maguire and Mody, 2007).

#### Neurosteroids

Endogenous neuroactive ligands synthetized from progesterone, deoxycorticosterone, or testosterone, referred to as neurosteroids interact with a number of targets, most notably GABA_A_ receptors and act as positive or negative allosteric modulators. Numerous preclinical evidence demonstrate that neurosteroids modulate the HPA axis and adaptive responses to stress exposure (Crowley and Girdler, 2014), and exert anxiolytic or antidepressant effects in rodent models (Khisti et al., 2000; Guidotti et al., 2001; Rodriguez-Landa et al., 2007). Specifically, the progesterone-derived neurosteroids, allopregnanolone, a potent positive allosteric modulator of both synaptic and extrasynaptic GABA_A_ receptors, were shown to rapidly modulate BDNF expression in the rat brain (Naert et al., 2007; Nin et al., 2011; Almeida et al., 2019), which could explain its fast onset for antidepressant responses. Allopregnanolone has been tested for the treatment of post-partum depression using a formulation developed by SAGE, referred to as brexanolone. The rationale for this study is based on the precipitous drop at the time of delivery of estrogen and progesterone, and consequently a drop in allopregnanolone resulting in a loss of this key positive allosteric modulator of GABA_A_ receptors and a withdrawal like effect. Brexanolone has been delivered intravenously and tested in two Phase II and Phase III trials. Due to its very promising results, it was recently granted a FDA Breakthrough Therapy Designation for the treatment of post-partum depression, and it has also been tested in placebo-controlled Phase III trials for the treatment of MDD (Kanes S. et al., 2017; Kanes S.J. et al., 2017; Meltzer-Brody et al., 2018; Wilkinson and Sanacora, 2018). Another compound, SAGE-217, an improved allopregnanolone formula with higher oral bioavailability and longer half-life, which can be used for once daily oral administration, successfully completed a Phase II study for MDD and also received a FDA Breakthrough Therapy Designation (Sage Therapeutics, 2017). In addition, ganaxolone (Marinus Pharmaceuticals), a neuroactive steroid that acts as a GABA_A_ positive allosteric modulator, was initially developed for the treatment of epilepsy and anxiety, and currently is under Phase II trials for post-partum depression (Wilkinson and Sanacora, 2018).

#### GABA_B_ Receptors Ligands

The first prototypical GABA_B_ receptor agonist, bacoflen, was synthetized in 1962 and it was an invaluable pharmacological tool that influenced studies that led to the characterization of GABA_B_ receptors in the 1980s. Years later, with the development of the first GABA_B_ receptor antagonists, phacoflen and saclofen, additional work has lead to the development of compounds that more specifically target GABA_B_ receptors. Given that the GABA_B_ receptor is a heterodimer of two subunits (GABA_B1_ and GABA_B2_), that GABA_B1_ has been reported to have several splice variants (mainly GABA_B1A_ and GABA_B1B_), and that GABA_B_ receptors are located both pre- and post-synaptically, pharmacological studies targeting these receptors report very challenging and complex results (Bowery et al., 1980, 1981; Kaupmann et al., 1997; Cryan and Kaupmann, 2005; Jacobson et al., 2018).

Preclinical studies suggest that GABA_B_ agonists, positive allosteric modulators, and antagonists can produce antidepressant effects; unfortunately, there are very few clinical studies due to the lack of compounds adequate for human testing (Alexander, 2017). In rats, acute administration (i.p.) of baclofen or SKF97541, both GABA_B_ receptors agonists, or CGP7930, a GABA_B_ positive allosteric modulator, induced antidepressant-like effects in the forced swim test, whereas chronic administration increased the escape failures in the learned helplessness test (Nakagawa et al., 1999; Frankowska et al., 2007). However, other studies failed to find significant effects for agonists or positive allosteric modulators (Nakagawa et al., 1999; Slattery et al., 2005; Nowak et al., 2006). In humans, one study reported that bacoflen intensified depressive symptoms in MDD patients (Post et al., 1991). Studies of GABA_B_ antagonists have yielded more consistent results, with a large range of studies showing antidepressant-like effects induced by several different compounds administered either acute- or chronically, such as CGP36742 (also known as SGS742), CGP51176, CGP51176A, CGP56433A, and SCH50911 (Bittiger et al., 1993; Nakagawa et al., 1999; Mombereau et al., 2004; Slattery et al., 2005; Nowak et al., 2006; Frankowska et al., 2007). Interestingly, CGP36742 decreased learned helplessness behavior in rats (Nakagawa et al., 1999) and increased BDNF and NGF release in the cortex and hippocampus (Heese et al., 2000), as well as increased extracellular glutamate and SST in the rat hippocampus (Nyitrai et al., 1999; Nyitrai et al., 2003). Notably, this is the first GABA_B_ receptor antagonist that underwent clinical trials for cognition-enhancing activity and improved attention in patients with mild cognitive impairment (Froestl et al., 2004). Thus, considering that: (i) MDD patients in general have an upregulation of GABA_B_ receptors; (ii) genetic deletion of GABA_B_ receptors produce antidepressant-like effects; (iii) GABA_B_ receptors are implicated in the antidepressant actions of fast agents such as ketamine (Workman et al., 2013, 2015; Rosa et al., 2016); and (iv) GABA_B_ receptors antagonists offer a promising strategy for the development of novel fast-acting antidepressants, more studies and clinical trials are warranted to identify effective and safe agents.

### Neuropeptides

Because of the postmortem evidence of selective alterations of GABA interneuron subytpes, it is interesting to speculate on approaches to target the function of specific subpopulations of interneurons based on expression of selective neuropeptides. Preclinical studies demonstrate promising pharmacological evidence for two neuropeptides, NPY and SST, to treat MDD. Intraperitoneal or direct intracerebral (lateral ventricle, hippocampus, amydgala, or septum) infusions of the SST peptide or small molecule SST agonists induce anxiolytic- and antidepressant-like effects in naïve and chronically stressed rodents, as well as exert inhibitory feedback on the HPA axis (Engin et al., 2008; Engin and Treit, 2009; Yeung et al., 2011; Prevot et al., 2017). There are five (1–5) SST Gi-protein-coupled receptors that are distributed on SST-expressing GABAergic interneurons, and are mainly coupled with induction of K+ conductance leading to neuronal hyperpolarization (Jiang et al., 2003; Meis et al., 2005). The development of selective SST compounds and genetic approaches using specific SST receptor subtypes knockout animals suggest that SST2 receptor, the most abundant subtype in the brain, is a key target receptor for the antidepressant effects of SST (Viollet et al., 2000; Engin and Treit, 2009; Prevot et al., 2017). However, the plasma half-life of SST is very short making it unsuitable for clinical trials (Pinter et al., 2006; Engin and Treit, 2009).

Two more stable analogs, octreotide and lanreotide, have been tested in clinical studies to treat a wide range of diseases, such as inflammation, tumor growth, and pain (De Jong et al., 1999; Hofland et al., 1992; Carlton et al., 2004; Pinter et al., 2006); although these drugs show a high affinity to SST2 receptors, they lack selectivity, and induce a broad spectrum of undesired effects in both periphery and CNS (Pawlikowski and Melen-Mucha, 2003). Drugs that act as selective SST2 receptor agonists, such as L-779,976, have never been tested in clinical trials. Thus, given that SST levels were reported to be lower in the brain of MDD patients and stressed rodents (Frye et al., 2003; Tripp et al., 2011), which can be normalized by monoaminergic drugs (Faron-Gorecka et al., 2016, 2018), and preclinical evidence that SST induces antidepressant-like effects, clinical studies testing the antidepressant potential of selective SST2 receptor analogs with longer half-life merit additional attention.

Early preclinical studies also provided evidence that central administration of NPY induces anxiolytic- and antidepressant-like effects (Heilig and Murison, 1987; Heilig et al., 1989, 1992; Pich et al., 1993; Broqua et al., 1995; Redrobe et al., 2002a,b), as well as promotes stress adaptation and resilience (Thorsell et al., 2000; Sajdyk et al., 2008; Yang et al., 2018). In the brain, at least four subtypes of Gi-coupled receptors for NPY were identified (Y1, Y2, Y4, and Y5) (Larhammar and Salaneck, 2004) and the antidepressant-like effects of NYP are suggested to be mediated by Y1R (Redrobe et al., 2002a; Karlsson et al., 2008). Interestingly, NPY levels were decreased in treatment-resistant MDD patients (Heilig et al., 2004) and increased after treatment with SSRIs, an effect that was inversely correlated to depression severity (Nikisch et al., 2005). NPY administration in humans also represents a challenge due to short half-life, as well as undesired effects. To overcome this problem, clinical studies have focused on the therapeutic potential of intranasal NPY administration (Lacroix and Mosimann, 1996; Lacroix et al., 1996; Hallschmid et al., 2004, 2003). While a recent randomized dose-ranging study found that intranasal NPY is effective for the treatment of posttraumatic stress disorder with reduced side effects (Sayed et al., 2018), the antidepressant efficacy of intranasal NPY in MDD patients has not been tested. Given that Y1 agonists or Y2 antagonists also show promise in preclinical studies as antidepressants (Redrobe et al., 2002b), further studies of selective drugs as well as intranasal administration of NPY in MDD patients are warranted.

### Cannabinoid Agents

Endocannabinoids, such as anandamide and 2-arachidonoylglycerol, are pivotal endogenous neuromodulators that control GABA and glutamate release in the brain, mainly through actions on cannabinoid type 1 (CB_1_) and cannabinoid type 2 (CB_2_) receptors (although some endocannabinoids can also activate transient receptor potential vanilloid type 1 receptors) (Fogaça et al., 2012). CB_1_ and CB_2_ are G_i/o_-coupled receptors mostly located pre-synaptically, and their activation results in hyperpolarization and reduction of neurotransmitter release (Szabo and Schlicker, 2005). In the neocortex, CB_1_ receptors are expressed by multiple interneuron subpopulations, mostly in CCK-, but are also found in SST-, calbindin-, and VIP-expressing cells, and at lower levels in glutamatergic neurons (Hill et al., 2007; Wedzony and Chocyk, 2009). Given that (i) CB_1_ receptors are highly expressed in cortical and limbic regions (Pettit et al., 1998; Wang et al., 2003), (ii) CB_1_ receptors are expressed in cortical interneurons and glutamatergic pyramidal cells, thereby modulating both GABA and glutamate release (Hill et al., 2007), and (iii) endocannabinoids act as retrograde messengers to mediate depolarization-induced suppression of E (DSE) and I (DSI) (Diana and Marty, 2004; Hill et al., 2007), it is not surprising that the endocannabinoid system plays an important role in orchestrating cortical E:I balance and controlling stress responses. Indeed, in the mPFC, endocannabinoids contribute to the termination of HPA activity during stress responses through inhibition of GABA release, increasing the outflow of principal interneurons to target regions (Hill et al., 2011).

Cannabinoid agents have shown promise for the treatment of anxiety disorders and depression (Poleszak et al., 2018; Stampanoni Bassi et al., 2018). The most studied compound for therapeutic use is cannabidiol (CBD), the major non-psychotomimetic substance from *Cannabis sativa.* Although CBD has a low affinity for CB_1_ and CB_2_ receptors, it enhances endocannabinoid neurotransmission by interfering with the function of fatty acid amide hydrolase (FAAH), the enzyme responsible for anandamide degradation (Bisogno et al., 2001; De Petrocellis et al., 2011; Fogaça et al., 2018). Also, CBD acts as an allosteric modulator of 5HT_1A_ receptors and was recently shown to exert direct actions at GABA_A_ receptors (Russo et al., 2005; Bakas et al., 2017). Accumulating clinical and pre-clinical evidence suggests that acute and chronic administration of CBD induces anxiolytic and antidepressant effects, as well as prevents the behavioral consequences of CUS (Schiavon et al., 2016; Campos et al., 2017; Crippa et al., 2018; Fogaça et al., 2018; Sales et al., 2018). Interestingly, the rapid molecular changes induced by CBD are similar to several glutamatergic and GABAergic rapid-acting drugs discussed so far, whereas the long-term effects resemble monoaminergic drugs. For example, a single injection of CBD promotes synaptogenesis in the mPFC and induces rapid and sustained antidepressant effects through increased mTORC1/BDNF signaling (Sales et al., 2018), and repeated administration of CBD prevents the decrease in neuronal remodeling/function and hippocampal neurogenesis induced by CUS (Campos et al., 2013; Fogaça et al., 2018). In spite of these advances in the mechanism of action of CBD and other cannabinoid agents, there are very few studies that have investigated the role of the GABAergic system. Thus, more causal studies should be performed to determine the subtype of interneuron populations that mediate the anxiolytic and antidepressant effects of cannabinoid drugs, as well as other GABA-related cellular and synaptic mechanisms that could be involved.

## Conclusion and Future Directions

For decades, the monoaminergic deficit hypothesis of depression was the prevalent theoretical basis for studies of the mechanisms underlying the pathophysiology and treatment of depression. However, although increased extracellular monoamines underlies the acute actions of monoamingergic agents, altered monoamine levels alone in forebrain areas are insufficient to explain the molecular and cellular changes underlying the antidepressant actions of these agents. Moreover, there is little consensus evidence that depression results from a deficit of monoamines. Thus, research has focused on neurotransmitter systems and microcircuits that can explain both the efficacy of antidepressant drugs and the etiology of MDD. Given growing consensus that MDD patients have a decrease in GABA levels in the brain and the revolutionary discovery that NMDA receptors antagonists, such as ketamine, can produce rapid and sustained antidepressant responses, efforts have been made to link the deficits in amino acid neurotransmitter systems to the pathophysiology of depression. Notably, the GABA deficit and the imbalance of cortical E:I hypothesis of depression provide a broader understanding of depression, as it offers connections with other important conceptual frameworks, such as altered glutamate and neurotrophic factor deficit hypotheses. With recent advances and new approaches, researchers have renewed enthusiasm for the development of fast-acting antidepressants that target the GABAergic and glutamatergic systems and overcome current therapeutic limitations of monoaminergic drugs. Despite recent advances, significant challenges remain, including development of more selective GABA, NMDA, and neuropeptide receptor agonists, antagonists, and modulators, characterization of optimal doses and treatment schedules, and better design of clinical trials. Moreover, genetic, chemogenetic, and optogenetic approaches should be directed to elucidate the role of specific interneuron subtypes and mechanisms underlying the control of behaviors related to mood and emotion, as well as sex-specific differences involved in these processes, with a view to developing more selective and improved antidepressant treatments.

## Author Contributions

MF designed and wrote the manuscript, revised the literature, and prepared the figure and table. RD revised, edited, and approved the manuscript, figure, and table, and contributed in writing the manuscript.

## Conflict of Interest Statement

The authors declare that the research was conducted in the absence of any commercial or financial relationships that could be construed as a potential conflict of interest.
